# OA13 Multiple complication in the lifetime of limited cutaneous systemic sclerosis

**DOI:** 10.1093/rap/rkac066.013

**Published:** 2022-09-28

**Authors:** Hoda Alkoky, Frances Hall

**Affiliations:** Cambridge University Hospital, Cambridge, United Kingdom; Cambridge University Hospital, Cambridge, United Kingdom

## Abstract

**Introduction/Background:**

We are reporting the case of a 76 year old woman whose symptoms started early in her teens with Raynaud's disease and who was first diagnosed with limited cutaneous systemic sclerosis in her twenties, at which time she had calcinosis, Raynaud's phenomenon, sclerodactyly and a drawstring mouth. She was started on vasodilators and remained relatively stable for several years.  However, she developed a series of complications from the late 1990s. onwards. These included digital ulceration, macrovascular disease, scleroderma bowel, pulmonary hypertension and severe calcinosis.

Comorbidities included ischemic heart disease, aortic stenosis and hypothyroidism.

She smoked for 5 years (19-24y).

**Description/Method:**

Dominant clinical problems

1980s Digital Ulceration

Started in her forties. She was treated with a series of vasodilators including nifedipine, losartan, fluoxetine and, from 2015, sildenafil. She required IV iloprost three monthly since 2004. Bosentan was considered but cautioned against, given also diagnosed with aortic stenosis.

1980s Calcinosis

Recurrent calcinotic fingers and the olecranon processes with cellulitis and no osteomyelitis.

1999-2007 Critical ischemia of legs

In 1999, she developed left leg ischemia.  Doppler ultrasound demonstrated severe stenosis, or occlusion, of all lower limb arteries. She was taking Nifedipine, Amlodipine, Losartan, Dipyridamole, Atorvastatin, Sildenafil, monthly courses of iloprost and opiate analgesia. Despite this, she proceeded to below-knee amputation in 2000. In 2006 she developed right leg ischemia and, despite transient improvement with posterior tibial angioplasty, she required below-knee amputation in 2007.

1999-2021 Iron deficiency anaemia

Iron deficiency anaemia was detected in 1999 (Hb 109 g/L, serum iron 5.5 umol). OGD showed oesophagitis and residual gastric contents in the oesophagus. In 2021 (Hb 112g/L) a repeat OGD showed stomach and duodenal angiodysplastic lesions.

Treatment: esomeprazole and Nizatidine.

2017 Suspected Pulmonary Arterial Hypertension

She was referred to the Pulmonary Vascular Diseases Unit at Royal Papworth hospital due to suspicion of pulmonary arterial hypertension following screenings:

· Lung Function: FEV1 99.8%, FVC 114.9% TLCOc 56%, KCOc 67%.

· ECHO LVEF 60%, normal R ventricle, normal atria, calcification of mitral valve annulus, mild tricuspid regurgitation with estimated sPAP 20-25 mmHg + RAP.

· Bloods: Uric acid 180 micromol/L, NT-proBNP 321pg/ml; FBC normal Pulmonary hypertension was confirmed.

· CTPA: Pulmonary emboli in right lower lobe segmental and subsegmental arteries.

· Right Heart Catheterisation: mPAP 29mmHg, PAWP 7mmHg, PVR 381 dynes (Test performed after withholding Sildenafil for 5 days)

Diagnosis: Chronic thromboembolic pulmonary hypertension with possible contribution from pulmonary arterial hypertension.

Treatment:  Warfarin and Sildenafil increased to 50mg tds.

**Discussion/Results:**

This patient developed both micro- and macrovascular disease. Digital ulcers were managed with escalation of vasodilators but bosentan was not added to sildenafil due to aortic stenosis. Macrovascular disease was treated with statins, angioplasty and, ultimately with amputation, she walks with two prosthetic legs and a stick.  It is unclear whether her short smoking history is relevant.

Iron deficiency anaemia can occur due to malabsorption due to scleroderma bowel but, in this case, recurrent bleeding from gut telangectasiae seemed most likely.

Pulmonary arterial hypertension is a well-recognised complication of systemic sclerosis. However, it is important that, the underlying cause(s) of pulmonary hypertension are investigated in order to manage appropriately.

Calcinosis can be debilitating and a cause of ulceration and infection. There are limited treatment options.

**Key learning points/Conclusion:**

Management of digital ulceration, with escalation of treatment and position of Sildenafil and Bosentan in treatment pathway.

Importance of considering macrovascular disease in systemic sclerosis.

Consideration of reasons for iron deficiency anaemia in systemic sclerosis.

Screening for and diagnosis of distinct types of pulmonary hypertension.

Limited management strategies for calcinosis.

